# Infarct growth precedes cerebral thrombosis following experimental stroke in mice

**DOI:** 10.1038/s41598-021-02360-6

**Published:** 2021-11-24

**Authors:** Vanessa Göb, Maximilian G. Voll, Lena Zimmermann, Katherina Hemmen, Guido Stoll, Bernhard Nieswandt, Michael K. Schuhmann, Katrin G. Heinze, David Stegner

**Affiliations:** 1 Institute of Experimental Biomedicine I, University Hospital Würzburg, Würzburg, Germany; 2 Rudolf Virchow Center for Integrative and Translational Bioimaging, University of Würzburg, Würzburg, Germany; 3 Department of Neurology, University Hospital Würzburg, Würzburg, Germany

**Keywords:** Cerebrovascular disorders, Thrombosis

## Abstract

Ischemic stroke is among the leading causes of disability and death worldwide. In acute ischemic stroke, successful recanalization of occluded vessels is the primary therapeutic aim, but even if it is achieved, not all patients benefit. Although blockade of platelet aggregation did not prevent infarct progression, cerebral thrombosis as cause of secondary infarct growth has remained a matter of debate. As cerebral thrombi are frequently observed after experimental stroke, a thrombus-induced impairment of the brain microcirculation is considered to contribute to tissue damage. Here, we combine the model of transient middle cerebral artery occlusion (tMCAO) with light sheet fluorescence microscopy and immunohistochemistry of brain slices to investigate the kinetics of thrombus formation and infarct progression. Our data reveal that tissue damage already peaks after 8 h of reperfusion following 60 min MCAO, while cerebral thrombi are only observed at later time points. Thus, cerebral thrombosis is not causative for secondary infarct growth during ischemic stroke.

## Introduction

Stroke is the second leading cause of death and disability worldwide and mostly caused by thromboembolic occlusion of major brain vessels. In human stroke the middle cerebral artery (MCA) and its branches are most commonly affected and account for approximately 70% of infarcts^1^. There are several models available to study ischemic stroke in animals. The model of transient middle cerebral artery occlusion (tMCAO) affects the MCA territory and is very close to the human situation and therefore widely used in experimental stroke research^2,3^. For the therapy of acute ischemic stroke, recanalization of the occluded vessel is essential, but not sufficient to guarantee brain salvage^4^. The phenomenon of ongoing ischemic lesion development despite recanalization is referred to as *reperfusion injury.* Often, abnormalities in cerebral blood perfusion lead to unfavorable outcomes as post-stroke hypoperfusion is associated with infarct expansion^5^. In the tMCAO model, complete recanalization is achieved by the removal of the filament, however cerebral perfusion is still not restored to 100% but only to approximately 50%^6^. It is well known that platelets contribute to ischemia/reperfusion injury of the brain^7^, but it is still controversially discussed whether ongoing tissue damage is caused by cerebral microthrombosis or not. Thrombotic activity after recanalization is still widely seen as one cause for the breakdown of the microcirculation, incomplete reperfusion and subsequent tissue death^8–10^. However, the fact that complete blockade of platelet aggregation via anti-GPIIb/IIIa treatment was not beneficial in either mice^11^ or patients^12,13^, suggests that thrombus formation is an unlike reason for tissue death. To clarify this, we examined the time course of infarct progression, platelet deposition and thrombus formation in the murine brain after tMCAO and confirmed that the occurrence of cerebral thrombi correlated with infarct size after 24 h but was secondary to neuronal damage.

## Methods

### Animals

Animal experiments were performed following the regulations of the National Institute of Heath Guidelines for the Care and Use of Laboratory Animals in compliance with ARRIVE guidelines^14^ and the approval of local authorities (District government of Lower Franconia). To reduce variability and thereby mouse numbers, only male mice with an age of 10–14 weeks were used. Recent studies showed remarkable effects of sex differences on the inflammatory response and infarct sizes^15^. Assuming a reduction of infarct volume of 30% as functionally relevant and a standard deviation of 20% to the respective mean values, a group size of ≥ 8 was necessary to show this effect with a power of 0.8 and a probability of a type I error of < 0.5 (calculated with GraphPad StatMate 2.00). The distribution of the mice into the different groups was randomized using research randomizer (randomizer.org).

### Transient middle cerebral artery occlusion

10- to 14-week-old, male, C57BL/6 J mice were applied to the tMCAO model and infarct sizes were determined as previously described^6^. Mice were anesthetized by 2% isoflurane inhalation anesthesia. A silicon rubber-coated filament (6021PK10, Doccol, Redlands, CA) was inserted in the carotid artery and advanced up to the origin of the middle cerebral artery (MCA). Surgical procedure was kept below 10 min for each mouse. After 60 min, the filament was removed allowing reperfusion. Animals were sacrificed after the indicated reperfusion times and brains were removed for further analysis. Exclusion criteria were death of the animals following tMCAO or signs of subarachnoidal hemorrhage (SAH) noted during brain sampling.

### Infarct size measurement

Removed brains were cut into three 2 mm thick coronal sections and viable tissue was stained with 2% 2,3,5 triphenyltetrazolium chloride (TTC; Sigma-Aldrich) for 20 min at 37 °C. Brain slices were scanned, and edema-corrected infarct volumes were calculated by planimetry (ImageJ Software, National Institutes of Health) with the following equation: V_indirect_ (mm^3^) = V_infarct_ × (1 − (VI − VC)/VC). VI: Volume ischemic hemisphere, VC: Volume control hemisphere.

### Immunohistochemistry

After staining of infarct volumes, the middle brain segment was cryo-embedded and cut into 10 µm thick slices. For the quantification of occluded vessels, brain slices were stained with hematoxylin and eosin. The number of occluded and open vessels was counted from the blinded samples under 20-fold magnification in the ischemic hemisphere and the percentage of occluded vessels was calculated.

For quantification of the number of platelet rich thrombi, platelets were stained with AF594-conjugated anti-platelet GPIX derivative^16^ (5 µg/ml); nuclei were stained with DAPI. Sections were imaged using a Leica SP8 confocal microscope and the whole brain section was recorded. Thrombi were quantified from tile scans using the Analyze Particle tool in Fiji (ImageJ Software, National Institutes of Health). Every “particle” larger than 5 µm^2^ was counted as thrombus.

### Western blot

Protein extraction and Western blot analysis were performed according to standard procedures and as previously described^17^. Dissected cortices were homogenized in RIPA buffer containing protease inhibitor cocktail (cOmplete™ protease inhibitor cocktail, Sigma-Aldrich) and sonified for 10 s. Residual tissue was removed via centrifugation at 15.000 g for 30 min at 4 °C. After determination of the protein content in the supernatant via bicinchoninic acid (BCA), lysates were used for Western Blot analysis. Anti-mouse GPIbβ antibody p0p1^18^ (10 µg/ml) was used for the quantification of platelet protein in the brain tissue. Actin (anti-Actin, A5441, Merck; 1:250,000) was used as loading control and for normalization of protein content.

### Light sheet fluorescence microscopy (LSFM)

30 min before the end of the desired reperfusion time platelets and the vasculature were stained by intravenous injection of AF750-conjugated anti-GPIX derivative (0.6 µg/g body weight)^19^, AF647-conjugated anti-CD105^20^ and AF647-conjugated anti-CD31 (each 0.4 µg/g body weight; BioLegend). 30 min after in vivo labeling (= indicated reperfusion times), mice were anesthetized by intraperitoneal injection of medetomidine 0.5 µg/g, midazolam 5 µg/g and fentanyl 0.05 µg/g body weight and transcardially perfused with ice-cold PBS and ice-cold 4% paraformaldehyde (PFA, Sigma-Aldrich, Schnelldorf, Germany, pH 7.2). Brains were removed, dehydrated in methanol solutions of increasing concentrations (50%, 70%, 95%, 100%) for 8–12 h each at 4 °C and stored in 100% methanol overnight. For optical clearing, brains were incubated in BABB solution (2 parts benzyl benzoate mixed with 1 part benzyl alcohol; both Sigma-Aldrich) for 12 h and stored in fresh BABB solution for another 12 h before imaging. Optically cleared brains were imaged with a custom-build light sheet microscope equipped with two EC Epiplan. Neofluar 2.5x/0.06 M27 excitation objectives (Zeiss, Germany) and a HCX FLUOTAR 5x/0.15 Dry detection objective (Leica, Germany) as previously described^19^. Information about the analysis of the LSFM images is given in the supplemental methods.

### Statistical analysis

Normal distribution of the data was analyzed using D’Agostino & Pearson omnibus normality test. Since normality was not met by all subsets, statistical differences between two groups were analyzed using the non-parametric two-tailed Mann–Whitney U test. P-values < 0.05 were considered statistically significant and p-values are indicated in the figures. In all figures, results are indicated as mean ± SD.

## Results

**Figure 1:**
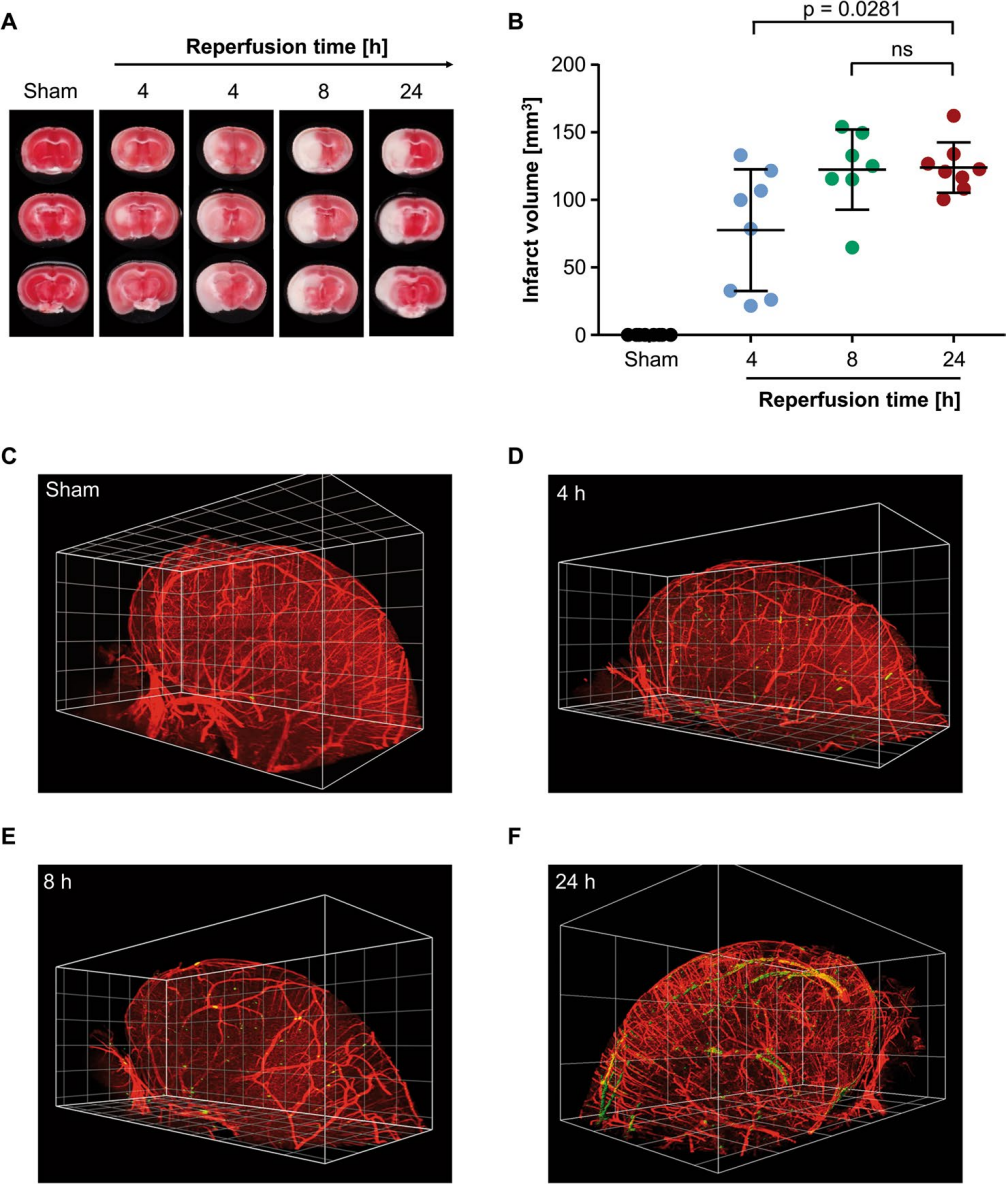
Cerebral thrombus formation occurs after development of infarcts following tMCAO. (**A**) Representative images of brain sections stained with TTC to visualize infarcts. Each timepoint shows three consecutive brain sections from one mouse. White: Infarct, Red: viable tissue. (**B**) Edema corrected quantification of infarct sizes. Data is plotted using GraphPad Prism 7.05 (https://www.graphpad.com) depicted as mean ± SD and each dot represents one mouse. N = 7–8 per time point. (**C**–**F**) 3D reconstruction of the ipsilateral hemispheres of mice after (**C**) sham surgery, (**D**) 4 h of reperfusion, (**E**) 8 h of reperfusion or (**F**) 24 h of reperfusion. Vessels (CD105/CD31; red), platelets (GPIX; green). 3D reconstructions were generated using Biptlane Imaris 9.6 (https://imaris.oxinst.com/); grid size: 0.5 mm. Statistical differences were analyzed using two-tailed Mann–Whitney U test. *P*-values < 0.05 were considered statistically significant. Compared to sham, differences to all other groups are highly significant with *P* < 0.001.

**Figure 2:**
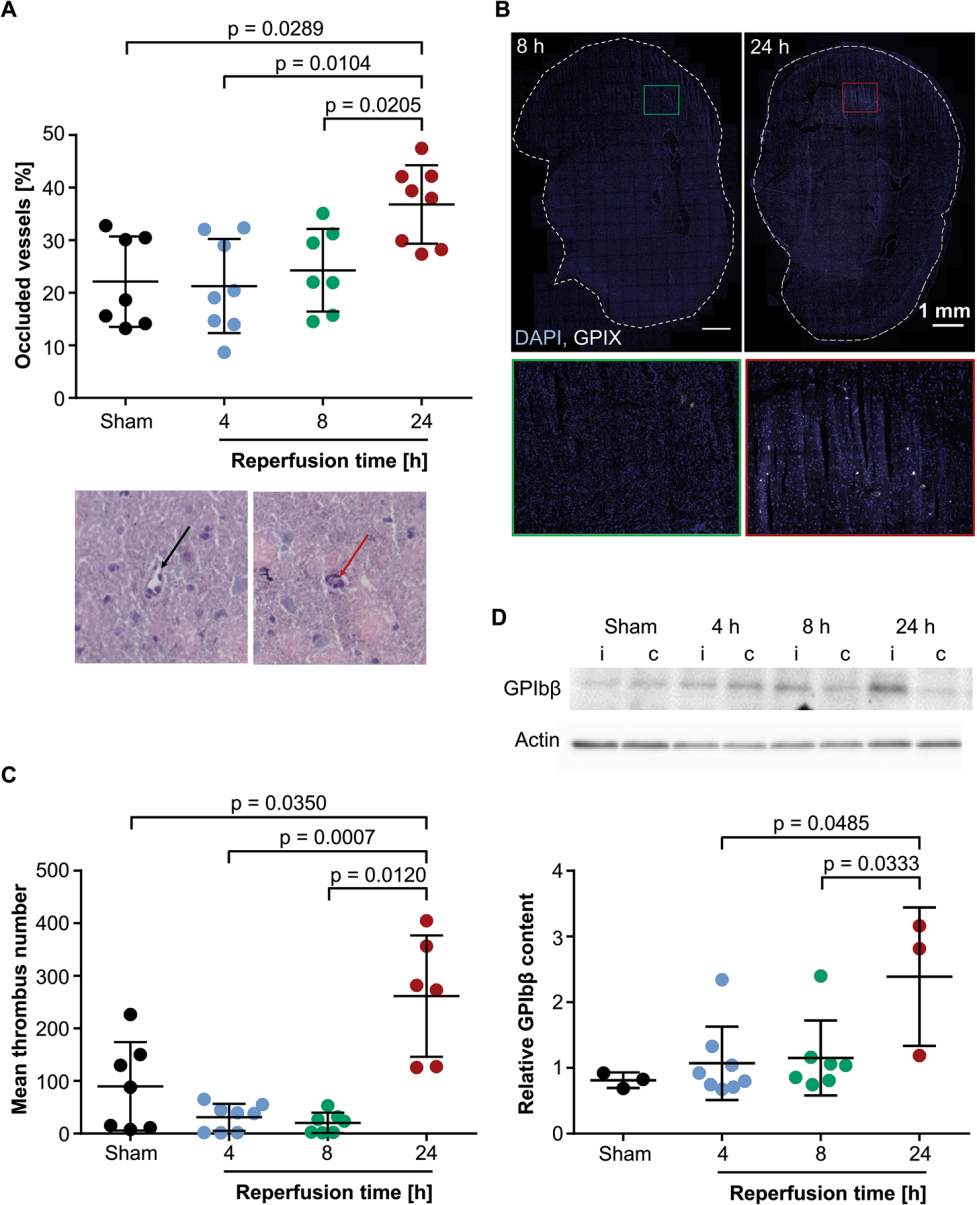
Cerebral thrombi are barely detectable within the first 8 h of reperfusion. (**A**) Quantification of occluded vessels in hematoxylin and eosin stained cryosections. Each dot represents the mean of 3–4 sections from one mouse. N = 7–8 per time point. Exemplary image of open (black arrow) and occluded (red arrow) vessel at 20 × magnification. (**B**) Representative z-projections of brain cryosections stained with anti-GPIX (platelets, white) and DAPI (cell nuclei, blue). Lower images show magnification of the respective rectangle in the brain section. (**C**) Quantification of thrombus number within brain cryosections. Each dot represents the mean of 3–4 sections from one mouse. N = 6–8 (**D**) Quantification of platelet GPIbβ protein (22 kDa) in brain lysates of the cortex. GPIbβ was normalized to actin (42 kDa) and is indicated as relative content. N = 3–8. I: ipsilateral; c: contralateral. Uncropped membrane can be found in Suppl. Fig. 1. Statistical differences were analyzed using two-tailed Mann Whitney U test. *P*-values < 0.05 were considered statistically significant and are indicated in the figures. In all panels, data is plotted using GraphPad Prism 7.05 (https://www.graphpad.com) and depicted as mean ± SD, each dot represents one mouse.

In our attempt to investigate the correlation of cerebral thrombosis and infarct progression we subjected mice to the tMCAO model with a standard occlusion time of 60 min and characterized the time course of infarct growth. After different reperfusion times, infarct sizes were determined. Infarcted tissue (white) can be seen first after 4 h of reperfusion (Fig. 1A). At this time point individual variance is the highest, as some mice show already large infarcts, whereas others do not (Figs. 1, 2nd and 3rd panel, Fig. 1B). After 8 h of reperfusion, however, infarcts have reached their maximum size (122.4 ± 29.65 mm^3^) and do not further increase with longer reperfusion time (infarct size after 24 h: 124.0 ± 18.67 mm^3^; Fig. 1A,B) indicating that processes leading to tissue death must occur within the first 8 h of the reperfusion phase.

To analyze the formation of thrombi in the time course of reperfusion we analyzed whole brain hemispheres in 3D using light sheet microscopy and developed an image processing and analysis strategy to quantify thrombi within the 3D reconstructions. In brains of sham-operated mice, almost no thrombi could be detected (25 ± 18; Fig. 1C, Suppl. Video 1). In brains that were removed after 4 h or 8 h of reperfusion, no significant increase in thrombus number was detected (98 ± 86 and 61 ± 25, respectively; Fig. 1D,E; Suppl. Videos 2, 3). Quantifying thrombus number in brains that were removed after 24 h of reperfusion revealed significantly elevated numbers (468 ± 463; Fig. 1F, Suppl. Video 4) and a clear difference can be seen in comparison to brains after 8 h of reperfusion, despite comparable infarct sizes (Fig. 1B). These data indicate that tissue death following experimental stroke precedes cerebral thrombus formation.

To exclude that the lack of cerebral thrombi is a result of perfusion artefacts during sample preparation for light sheet microscopy, we analyzed cryosections of brain segments that were removed at the indicated time points without prior perfusion fixation. We quantified the number of occluded vessels in hematoxylin and eosin-stained sections (Fig. 2A) and the number of thrombi in sections stained specifically with an anti-platelet antibody (Fig. 2B,C). After 24 h of reperfusion, the percentage of occluded vessels and the number of thrombi (36.83 ± 7.46% and 261.5 ± 115.4, respectively) are significantly higher than for sham operated mice (22.14 ± 8.59% and 89.74 ± 84.05) which correlates with the infarct sizes (Fig. 1B). Strikingly, at earlier time points, after 4 h and after 8 h of reperfusion, the number of occluded vessels (4 h: 21.29 ± 8.95%; 8 h: 24.29 ± 7.86%) as well as the number of thrombi (4 h: 30.89 ± 25.67; 8 h: 20.29 ± 19.45) were comparable to those of sham operated mice (Fig. 2A–C). Given the fact that infarct sizes have already reached their maximum after 8 h, the absence of thrombi at this time point indicates that cerebral microthrombi are not causative for infarct progression.

For an imaging independent quantification of platelets within the brain hemispheres, we performed Western Blot analysis of brain tissue and quantified the amount of GPIbβ, a platelet-specific protein (that is not released from activated platelets), within the cortical region of the brains. Here, the relative GPIbβ content in brain tissue of sham operated mice (0.81 ± 0.11), after 4 h (1.07 ± 0.55) or 8 h (1.15 ± 0.57) of reperfusion is similar and only after 24 h of reperfusion, GPIbβ content is significantly increased (2.39 ± 1.05; Fig. 2D). The contralateral hemisphere was taken as internal control and shows no increase in GPIbβ levels at any time point.

## Discussion

The primary therapeutic goal for acute ischemic stroke patients is the recanalization of the occluded vessel allowing reperfusion of the ischemic tissue. Despite restoration of blood flow, not all patients benefit, and further (‘secondary’) infarct growth can be observed^4^. The degree of cerebral perfusion after recanalization has been found to correlate with infarct progression^21^. Thrombi in the microcirculation, that either result from embolization of the initial stroke causing occlusion (during reperfusion) or emerge secondary within the cerebral vasculature, would be a plausible explanation for the insufficient reperfusion. In line with this, several studies showed a positive correlation of the occurrence of occluded vessels/thrombi with infarct sizes at day 1 after tMCAO^22–24^. However, interpretation of these data must be taken with care because this does not necessarily mean that vessel occlusion is the reason for infarct development. Nevertheless, these results have spurred the concept that thrombotic events during reperfusion trigger a breakdown of the microcirculation resulting in tissue damage and secondary infarct growth.

We have investigated the kinetics of infarct progression following experimental stroke and already after 4 h of reperfusion significant infarcts are developing. At this early time point after tMCAO the infarct sizes are variable (Fig. 1B), indicating that the infarct growth is not complete, while at 8 h and 24 h, all mice have big infarcts and no further increase in infarct size was observed between 8 and 24 h. Using several complementary approaches, we reveal here that thrombi can only be detected in the cerebral vasculature after infarct progression excluding thrombosis as cause for secondary infarct growth. We analyzed the number of occluded vessels using H&E staining (Fig. 2A) as surrogate marker for cerebral thrombosis and determined cerebral thrombi via immune fluorescence staining (Fig. 2B,C). In both cases, thrombi were also observed in the sham group, indicating limitations of those two methods, as some residual platelet activity might be triggered by the death of the animals. Furthermore, thin sections have the limitation of providing few information in the z-plane. In contrast to this, Western blotting (Fig. 2D), where normalization to the contralateral hemisphere and LSFM, which includes perfusion of the animals, have less ‘thrombotic activity’ in the sham group and provide information of the situation in the whole hemisphere. Notably, however, independently of the methodology used, no differences were observed between the sham group and the 4 h and 8 h reperfusion groups, while 24 h after reperfusion significantly more thrombi were observed.

The occurrence of thrombi in the infarcted brain tissue after tissue damage might play a dual role. On the one hand, thrombi might further decrease supply of the infarcted tissue and promote later stages of tissue death. On the other hand, thrombi are likely the consequence of ongoing tissue death in the infarcted brain areas. Tissue death liberates platelet activating mediators which promote thrombus formation in the affected areas. Upon activation, platelets release a variety of factors that are known to be involved in angiogenesis, immune-modulation and tissue repair^25^. Therefore, thrombus formation might be an active part of tissue regeneration. Further studies are warranted to investigate the role of platelets in tissue regeneration after stroke.

In conclusion, our data exclude cerebral thrombus formation as major cause for infarct progression during the reperfusion phase following tMCAO. Of note, these data are in line with the fact that blocking platelet aggregation by targeting GPIIb/IIIa failed in stroke therapy^12,13^.

Nevertheless, platelets contribute to reperfusion injury since absence of other platelet receptors or components has been shown to improve outcomes following tMCAO^7^. Furthermore, blocking initial platelet adhesion improves cerebral perfusion and reduces infarct sizes following experimental stroke^26^ and reduces tissue damage already during the occlusion phase^27^. This supports the concept, that the platelets’ role to infarct progression is different from ‘just’ thrombosis and evidence is given that this might be rather of immune-modulating nature^6,7,28^. Reperfusion injury is a complex phenomenon involving presumably a plethora of cells and molecules (reviewed in^29^). Collapse of the microcirculation can have various reasons, it might result from extrinsic compression and/or intravascular events^30^. So far, involvement of astrocytes, pericytes, endothelial cells, leukocytes and small molecules like nitric oxide or acetylcholine has been shown^31^. Platelets might directly contribute to tissue damage, or, potentially, recruit neutrophils, that have been shown to contribute to the no-reflow phenomenon following ischemic stroke^32,33^. Clearly, future studies are needed to elucidate step by step how platelets and other cell types contribute to the ongoing tissue damage following ischemic stroke.

## Supplementary Information

Supplementary Information 1.

Supplementary Video 1.

Supplementary Video 2.

Supplementary Video 3.

Supplementary Video 4.

## Data Availability

Any material/datasets generated in this study are available upon reasonable request from the corresponding authors.
