# Supplementary material for: Infarct growth precedes cerebral thrombosis following experimental stroke in mice
Source: Sci Rep. 2021 Nov 24;11:22887. doi: 10.1038/s41598-021-02360-6 (PMC8613266; doi:10.1038/s41598-021-02360-6)
Supplement: Supplementary file 1 — Supplementary Information 1. [file 41598_2021_2360_MOESM1_ESM.pdf]

# Infarct growth precedes cerebral thrombosis following experimental stroke in mice

Vanessa Göb[1,2], Maximilian G. Voll[1], Lena Zimmermann[3], Katherina Hemmen[2], Guido Stoll[3], Bernhard Nieswandt[1,2], Michael K. Schuhmann[3], Katrin G. Heinze[2*] and David Stegner[1,2*]

Running head: Cerebral thrombus formation in ischemic stroke

[1]Institute of Experimental Biomedicine I, University Hospital Würzburg, Würzburg, Germany

[2]Rudolf Virchow Center for Integrative and Translational Bioimaging, University of Würzburg, Würzburg, Germany

[3]Department of Neurology, University Hospital Würzburg, Würzburg, Germany

*Address correspondence to:

David Stegner: stegner@virchow.uni-wuerzburg.de

or Katrin G. Heinze: katrin.heinze@virchow.uni-wuerzburg.de

Keywords: ischemic stroke, thrombosis, platelet, reperfusion injury

# Supplemental Material

## Supplemental Methods

### Analysis of brain LSFM images

Images acquired by LSFM were saved in TIFF format and the thrombi and vessel channel were deconvolved using Huygens software (Scientific Volume Imaging (SVI), Hilversum, The Netherlands; signal-to-noise ratio: 40, background subtraction with nearest object within a 2 µm radius and 60 iterations). For object segmentation, firstly machine learning based pixel classification for thrombi was performed using the Ilastik [3] software. Next, binary images were exported based on the generated probabilities using the build-in simple segmentation option of Ilastik. These images of segmented thrombi were further processed using “Surface module” of the 3D rendering software Bitplane Imaris (Bitplane AG, Zürich, Switzerland) to detect platelet aggregates with a volume above 100 µm$^3$ and the total number of individual aggregates per brain was exported.

## Supplemental Figures

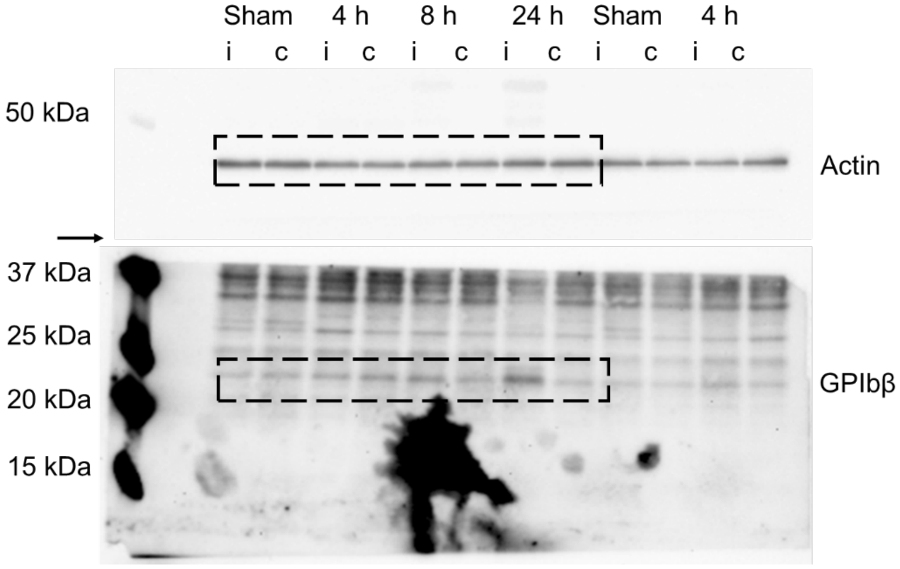


**Supplemental Figure 1. Quantification of platelet GPIbβ protein in brain lysates of the cortex**. Uncropped membrane of Western Blot shown in Figure 2D. Regions shown in Figure 2D are indicated by the black boxes. I: Ipsilateral; c: contralateral. The membrane was cut above the 37 kDa marker band (black arrow) before incubation with the different antibodies.

## Legends for supplemental video files

**Videos: Cerebral thrombi are only detectable after 24 h of reperfusion.** Animations of 3D reconstructions of ipsilateral brain hemispheres of **(1)** sham operated mice or mice after tMCAO with 1 h occlusion and **(2)** 4 h of reperfusion, **(3)** 8 h of reperfusion or **(4)** 24 h of reperfusion. Vessels (CD105/CD31; red), platelets (GPIX, green). Grid size: 0.5 mm.

## Supplemental References

1. Franke, M., et al., *The NLRP3 inflammasome drives inflammation in ischemia/reperfusion injury after transient middle cerebral artery occlusion in mice.* Brain Behav Immun, 2020.
2. Bergmeier, W., et al., *Structural and functional characterization of the mouse von Willebrand factor receptor GPIb-IX with novel monoclonal antibodies.* Blood, 2000. **95**(3): p. 886-93.
3. Berg, S., et al., *ilastik: interactive machine learning for (bio)image analysis.* Nat Methods, 2019. **16**(12): p. 1226-1232.
